# Use of the Relative Citation Ratio in Conjunction With H-Index to Promote Equity in Academic Orthopaedics

**DOI:** 10.5435/JAAOSGlobal-D-23-00080

**Published:** 2023-07-05

**Authors:** Isabel Herzog, Dhruv Mendiratta, Daniel F. Liggio, David B. Ahn, Michael Vosbikian, Neil K. Kaushal, Alice Chu

**Affiliations:** From the Rutgers New Jersey Medical School, Newark, NJ.

## Abstract

Quantification of a researcher's productivity relies on objective bibliometric measurements, such as the Hirsch index (h-index). However, h-index is not field and time-normalized and possesses bias against newer researchers. Our study is the first to compare the relative citation ratio (RCR), a new article-level metric developed by the National Institutes of Health, with h-index in academic orthopaedics.

**Methods:**

Academic orthopaedic programs in the United States were identified using the 2022 Fellowship and Residency Electronic Interactive Database. Available demographic and training data for surgeons were collected. RCR was calculated using the National Institutes of Health iCite tool, and h-index was calculated using Scopus.

**Results:**

Two thousand eight hundred twelve academic orthopaedic surgeons were identified from 131 residency programs. H-index, weighted RCR (w-RCR), and mean RCR (m-RCR) all significantly differed by faculty rank and career duration. However, while h-index and w-RCR varied between sexes (*P* < 0.001), m-RCR did not (*P* = 0.066), despite men having a longer career duration (*P* < 0.001).

**Discussion:**

We propose that m-RCR be used in conjunction with w-RCR or h-index to promote a fairer, comprehensive depiction of an orthopaedic surgeon's academic effect and productivity. Use of m-RCR may reduce the historic bias against women and younger surgeons in orthopaedics, which has implications in employment, promotion, and tenure.

Quantification of a researcher's productivity and effect in academia often relies on objective statistical analyses of their publications, known as bibliometrics. Bibliometrics are becoming increasingly prominent in medicine, with the number of presentations related to research effect at the Medical Library Association annual meetings increasing more than threefold between 2013 and 2017.^1^ Currently, the most commonly used metric of research productivity is the Hirsch index (h-index).^2^

H-index is an author-level metric which was proposed by Hirsch in 2005. It was described in the following way: “A scientist has index h if h of his or her Np papers have at least h citations each and the other (Np–h) papers have ≤ h citations each.” Over time, h-index has had shortcomings in fully showing one's academic productivity. For instance, the metric has an inherent bias toward authors with longer career durations.^3^ In addition, it does not accurately reflect research output, as shown with an author with 10 citations on 10 publications being equal to an author with 10 citations on 100 publications. Finally, this metric was originally designed for researchers of physics and chemistry but has informally become accepted for other academic specialties.^4^ In 2015, to overcome these limitations, the National Institutes of Health (NIH) developed the relative citation ratio (RCR).^5^ RCR calculates an article-level score by dividing a publication's total number of citations by the average number of citations received by NIH-funded publications within the same field and year.^5^ An article published in 2016 has more time to accrue citations than one published in 2022, so comparing each article with others within the same year is a critical time-normalizing factor. Furthermore, scientific fields do not make the same progress every year, so comparing articles with others within their respective fields allows for field normalization.^6^

Research productivity is a defining factor in academic institutions because it affects the progress made by the institution for securing funding and research grants.^7,8^ There is a need for objective clinical and educational metrics on which to base the quality of an academician, whether for university promotion or for grant applications.^9^ To the author's knowledge, there have been no studies that evaluate RCR in the entire field of orthopaedics. Rather than just providing an overview of this metric, our study attempts to compare RCR with the current standard, the h-index. Our study provides an analysis of sex, career duration, academic rank, subspecialty, and region between these two metrics.

## Methods

### Data Collection

The following study was a retrospective cross-sectional analysis of academic orthopaedic surgeons in the United States. Academic orthopaedic programs active in 2022 were identified using the Fellowship and Residency Electronic Interactive Database. Publicly available demographic and training data for surgeons were collected from institutional websites and Doximity. Doximity is the largest social networking service used by healthcare professionals, residents, and students. These variables consisted of sex, faculty rank, career duration, fellowships, and location of residency training. Career duration was calculated by subtracting the year of an orthopaedic surgeon's fellowship graduation, or residency graduation if no fellowship was completed, from the year of data collection (2022). Programs were geographically categorized as Northeast, Midwest, West, or South based on the US Census.

Assistant professor, associate professor, and full professor were the only levels of faculty rank included. Clinical-track faculty, such as instructors, clinical assistant professors, clinical associate professors, and clinical professors, were excluded. Research-exclusive faculty; professor emeriti; and affiliated, courtesy, voluntary, or visiting faculty were also excluded. Tenure-track professors could not be distinguished from clinical educator-track professors because of insufficient public information.

Because there is variability in productivity by subspecialty in orthopaedics, the decision to include fellowship as an exploratory variable was made. Orthopaedic surgeons were classified as having completed no fellowship or an adult reconstruction, foot and ankle, sports medicine, spine, trauma, pediatrics, hand and upper extremity, musculoskeletal oncology, or upper extremity reconstruction (shoulder or shoulder and elbow), or “other” fellowship. The other category consisted of arthroscopic surgery, limb lengthening, hip and joint preservation, minimally invasive spine, metabolic bone disease, nonhand plastic and reconstruction, and neuro-orthopaedics.

### Determination of Metrics

RCR was calculated using the NIH iCite tool (https://icite.od.nih.gov/), and h-index was calculated using Scopus. Derivatives of the RCR for individual authors are the mean RCR (m-RCR) and weighted RCR (w-RCR), which are the yearly average and cumulative sum, respectively, of RCR scores of articles produced by an individual author. m-RCR is a proxy for research effect while w-RCR is a proxy for research productivity.

To account for the discrepancies in search results based on author name in iCite, the Scopus author lookup feature was used. This feature can be used to confirm an author's identity by middle initial, current affiliation, past affiliations, city, and country. From this, PubMed Identifier (PMID) were exported from an author's profile into iCite to record m-RCR and w-RCR. This verified an author's publication history and promoted consistency of input data for h-index and RCR calculations.

### Statistical Analysis

Statistical analyses were conducted using IBM SPSS Version 28. The h-index, m-RCR, and w-RCR data were not normally distributed, so medians and interquartile ranges (IQRs) were recorded. The Kruskal-Wallis test was used to compare the h-index, m-RCR, and w-RCR medians by faculty rank, geographic region, career durations, subspecialty, and number of fellowships, followed by the Dunn post hoc test with Bonferroni correction (*α* = 0.05). The Mann-Whitney *U* test was used to compare the h-index, m-RCR, and w-RCR by sex. Test statistics are provided with each finding.

Subspecialty data was calculated by assigning orthopaedic surgeons into cohorts based on their completed fellowships. For every completed fellowship, a surgeon was assigned a “1” in that subspecialty column. All the column 1 values were tabulated to create multiple subspecialty cohorts and assess academic productivity.

The 75th percentile of m-RCR scores, referred to as “top m-RCR,” was calculated for a binary logistic regression model. Bivariate χ2 goodness of fit was used to assess differences in distribution of demographic variables between “top m-RCR” and “non-top m-RCR” surgeons. Variables associated with a *P*-value of < 0.05 were advanced to a definitive logistic binary regression analysis of top m-RCR data. The following variables were included in this definitive analysis: faculty rank, career duration, and geographical region. Odds ratios were calculated for these variables, and confidence intervals (CIs) were calculated to 95% confidence. Multivariate analyses were only conducted for orthopaedic surgeons for whom all data points were available. Of the 2812 surgeons who were identified, 233 had at least 1 missing data point (including 233 with a missing m-RCR). Therefore, 2579 surgeons were included in this multivariate analysis.

## Results

### Demographics of Academic Orthopaedic Surgeons

Two thousand eight hundred twelve academic orthopaedic surgeons in the United States were identified from 131 residency programs. Table 1 summarizes demographic information about these academic orthopaedic surgeons. The median career duration in the cohort was 14 years (IQR, 7 to 25).

**Table 1:** Demographics of Academic Orthopaedic Surgeons in the United States (N = 2812)

Characteristic	No. of Surgeons	Percent
Sex		
Male	2452	87.2
Female	359	12.8
Faculty rank		
Assistant professor	1334	47.4
Associate professor	721	25.6
Full professor	757	26.9
Region		
Northeast	828	29.4
Midwest	477	17.0
West	445	15.8
South	1062	37.8
Years in practice		
0-10	1092	38.8
11-20	774	27.5
21-30	552	19.6
31+	394	14.0
Subspecialty^a^		
Adult reconstruction	376	13.4
Foot and ankle	217	7.7
Sports medicine	526	18.7
Spine	335	11.9
Trauma	405	14.4
Pediatrics	353	12.6
Hand and upper extremity	360	12.8
Musculoskeletal oncology	164	5.8
Shoulder and elbow	138	4.9
Other	70	2.5
No. of fellowships		
0	166	5.9
1	2242	79.7
2	317	11.3
3+	87	3.1

a Subspecialty percentage represents the number of orthopaedic surgeons who completed specific fellowship of 2812.

### Demographic and Career Differences in h-Index, w-RCR, and m-RCR

Tables 2–4 contain the medians and IQRs of h-index, w-RCR, and m-RCR, respectively, stratified by sex, faculty rank, region, career duration, subspecialty, and number of fellowships. The h-index was not available for 206 surgeons, and RCR was not available for 233 surgeons.

**Table 2:** h-Index of Academic Orthopaedic Surgeons Stratified by Demographic Characteristics

Characteristic	No. of Surgeons	h-Index^a^		*P* Value
		Median	IQR	
Sex				<0.001
Male	2274	10	4-20	
Female	331	6	3-12	
Faculty rank				< 0.001
Assistant professor	1178	5	2-9	
Associate professor	685	10	5-16	
Full professor	743	23	14-35	
Region				< 0.001
Northeast	771	11	5-21	
Midwest	444	10	5-20	
West	418	12.5	5-23	
South	973	7	3-15	
Years in practice				<0.001
0-10	1008	6	3-11	
11-20	723	10	5-19	
21-30	504	15	6-26	
31+	371	20	10-34	
Subspecialty				<0.001
Adult reconstruction	351	11	5-19	
Foot and ankle	200	9	4-17	
Sports medicine	491	11	4-22	
Spine	321	12	5-25	
Trauma	374	8.5	4-16	
Pediatrics	326	8	3-15	
Hand and upper extremity	335	8	4-15	
Musculoskeletal oncology	157	11	6-20	
Shoulder	133	10	4.5-19.5	
Other	63	13	6-22	
No. of fellowships				<0.001
0	137	8	2-21	
1	2089	9	4-19	
2	299	12	5-20	
3+	81	12	7-24	

h-index = Hirsch index, IQR = interquartile range.

a The h-index was not available for 206 surgeons who were included in the study.

**Table 3:** w-RCR of Academic Orthopaedic Surgeons Stratified by Demographic Characteristics

Characteristic	No. of Surgeons	w-RCR^a^		*P* Value
		Median	IQR	
Sex				0.001
Male	2255	29.75	7.68-79.52	
Female	324	14.13	3.97-36.10	
Faculty rank				0.001
Assistant professor	1157	10.17	3.21-28.88	
Associate professor	683	29.37	9.68-66.65	
Full professor	739	80.50	40.12-136.18	
Region				0.001
Northeast	757	33.96	10.50-87.64	
Midwest	441	29.05	8.71-75.60	
West	417	40.49	10.93-93.50	
South	964	16.89	3.98-56.17	
Years in practice				0.001
0-10	996	15.18	4.53-39.22	
11-20	718	29.03	7.79-71.82	
21-30	500	44.95	12.93-101.61	
31+	365	69.14	24.49-123.77	
Subspecialty				0.001
Adult reconstruction	347	35.00	10.80-101.61	
Foot and ankle	198	21.86	5.50-72.45	
Sports medicine	484	30.63	6.93-80.23	
Spine	318	41.20	9.42-91.54	
Trauma	371	24.63	7.20-65.67	
Pediatrics	326	17.78	4.95-52.03	
Hand and upper extremity	331	20.22	5.75-52.57	
Musculoskeletal oncology	155	28.61	9.75-74.17	
Shoulder	131	32.86	11.04-75.02	
Other	63	38.06	16.74-96.94	
No. of fellowships				0.003
0	135	18.16	2.96-80.45	
1	2066	25.11	6.73-72.94	
2	298	35.33	12.27-79.66	
3+	80	33.15	10.93-89.76	

IQR = interquartile range, w-RCR = weighted relative citation ratio.

a The w-RCR was not available for 233 surgeons who were included in the study.

**Table 4:** m-RCR of Academic Orthopaedic Surgeons Stratified by Demographic Characteristics

Characteristic	No. of Surgeons	m-RCR^a^		*P* Value
		Median	IQR	
Sex				0.066
Male	2255	1.63	1.14-2.21	
Female	324	1.47	0.95-2.25	
Faculty rank				0.001
Assistant professor	1157	1.49	0.90-2.14	
Associate professor	683	1.56	1.13-2.06	
Full professor	739	1.83	1.34-2.40	
Region				0.001
Northeast	757	1.69	1.20-2.24	
Midwest	441	1.60	1.14-2.32	
West	417	1.77	1.26-2.34	
South	964	1.50	0.96-2.06	
Years in practice				0.001
0-10	996	1.54	1.03-2.13	
11-20	718	1.59	1.07-2.10	
21-30	500	1.70	1.20-2.30	
31+	365	1.84	1.29-2.47	
Subspecialty				0.001
Adult reconstruction	347	1.93	1.36-2.52	
Foot and ankle	198	1.64	1.06-2.24	
Sports medicine	484	1.88	1.20-2.54	
Spine	318	1.81	1.23-2.39	
Trauma	371	1.53	1.07-2.03	
Pediatrics	326	1.33	0.95-1.77	
Hand and upper extremity	331	1.41	0.97-1.79	
Musculoskeletal oncology	155	1.26	0.88-1.80	
Shoulder	131	1.81	1.44-2.25	
Other	63	1.84	1.30-2.50	
No. of fellowships				0.272
0	135	1.58	0.99-2.32	
1	2066	1.60	1.10-2.18	
2	298	1.69	1.21-2.29	
3+	80	1.56	1.00-2.15	

IQR = interquartile range, m-RCR = mean relative citation ratio.

a The m-RCR was not available for 233 surgeons who were included in the study.

Men had a higher median h-index than women (10 vs. 6). The Mann-Whitney *U* test demonstrated significant differences in median h-indices between sexes (*U* = 278,872; *P* < 0.001). Men had a higher median w-RCR than women (29.75 vs. 14.13). The Mann-Whitney *U* test demonstrated significant differences in median w-RCR between sexes (*U* = 279,580; *P* < 0.001). Men had a higher median m-RCR than women (1.63 vs. 1.47). However, the Mann-Whitney *U* test demonstrated no significant differences in median m-RCR between sexes (*U* = 342,304.5; *P* = 0.066). The median career duration among men (15 [IQR, 8 to 26]) was higher than women (9 [IQR, 5 to 15]). The Mann-Whitney *U* test demonstrated significant differences in median career duration between sexes (*U* = 304,271.5; *P* < 0.001).

The median h-index was lowest for those in practice for 0 to 10 years and increased for each subsequent bracket (6 vs. 10 vs. 15 vs. 20). The Kruskal-Wallis test demonstrated significant differences in median h-index among career duration brackets (h = 400.09; *P* < 0.001). The median w-RCR was lowest for those in practice for 0 to 10 years and increased for each subsequent bracket (15.18 vs. 29.03 vs. 44.95 vs. 69.14). The Kruskal-Wallis test demonstrated significant differences in median w-RCR among the career duration brackets (h = 264.78; *P* < 0.001). The median m-RCR was lowest for those in practice for 0 to 10 years and increased for each subsequent bracket (1.54 vs. 1.59 vs. 1.70 vs. 1.84). The Kruskal-Wallis test demonstrated significant differences in median m-RCR among career duration cohorts (h = 35.00; *P* < 0.001). On post hoc analysis, there was no difference in median m-RCR between those in practice for 0 to 10 years and 11 to 20 years or those in practice for 11 to 20 years and 21 to 30 years. Only 31+ years was markedly greater than all other years in practice cohorts.

Full professors had the highest median h-index, followed by associate professors and assistant professors (23 vs. 10 vs. 5). The Kruskal-Wallis test demonstrated significant differences in median h-indices within faculty ranks (h = 934.45; *P* < 0.001). Full professors had the highest median w-RCR, followed by associate professors and assistant professors (80.50 vs. 29.37 vs. 10.17). The Kruskal-Wallis test demonstrated significant differences in median w-RCR within faculty ranks (h = 732.17; *P* < 0.001). Full professors had the highest median m-RCR, followed by associate professors and assistant professors (1.49 vs. 1.56 vs. 1.83). The Kruskal-Wallis test demonstrated significant differences in median m-RCR within faculty ranks (h = 78.86; *P* < 0.001). On post hoc analysis, there was no difference in assistant and associate professors in median m-RCR.

The Kruskal-Wallis test demonstrated significant differences in median h-index for regions (h = 103.58; *P* < 0.001), subspecialties (h = 58.25; *P* < 0.001), and number of fellowships (h = 16.39; *P* < 0.001). The Kruskal-Wallis test demonstrated significant differences in median w-RCR for regions (h = 102.87; *P* < 0.001), subspecialties (h = 63.11; *P* < 0.001), and number of fellowships (h = 14.32; *P* = 0.003). The Kruskal-Wallis test demonstrated significant differences in median m-RCR for regions (h = 39.91; *P* < 0.001) and subspecialties (h = 203.31; *P* < 0.001). The Kruskal-Wallis test demonstrated no significant differences in median m-RCR between the number of fellowships (h = 3.90; *P* = 0.272).

### Determinants of High m-RCR

The 75th percentile m-RCR score (top m-RCR) was calculated to be 2.21 or higher. In the cohort, 652 orthopaedic surgeons (23.2%) achieved top m-RCR. No significant differences were observed in the achievement of top m-RCR between sexes (χ2[1, N = 2812] = 0.028, *P* = 0.868). Full professors were 1.77 times more likely to achieve top m-RCR than assistant professors (95% CI, 1.45 to 2.18; *P* < 0.001). Northeastern orthopaedic surgeons were 1.30 times more likely to achieve top m-RCR than Southern orthopaedic surgeons (CI, 1.04 to 1.62; *P* = 0.021). The top m-RCR results are summarized in Table 5.

**Table 5:** Top Quartile m-RCR^a^ Scorers Stratified by Demographic Characteristics

Characteristic	<75th Percentile (%)	≥75th Percentile (%)	*P* Value
Sex			0.868
Male	1883 (76.8)	570 (23.2)	
Female	277 (77.2)	82 (22.8)	
Faculty rank			<0.001
Assistant professor	1061 (79.5)	273 (20.5)	
Associate professor	584 (81.0)	137 (19.0)	
Full professor	515 (68.0)	242 (32.0)	
Region			<0.001
Northeast	630 (76.1)	198 (23.9)	
Midwest	347 (72.7)	130 (27.3)	
West	326 (73.3)	119 (26.7)	
South	857 (80.7)	205 (19.3)	
Years in practice			<0.001
0-10	863 (79.0)	229 (21.0)	
11-20	618 (79.8)	156 (20.2)	
21-30	408 (73.9)	144 (26.1)	
31+	271 (68.8)	123 (31.2)	
No. of fellowships			0.415
0	128 (77.1)	38 (22.9)	
1	1731 (77.2)	511 (22.8)	
2	232 (73.2)	85 (26.8)	
3+	69 (79.3)	18 (20.7)	

m-RCR = mean relative citation ratio.

a The m-RCR was not available for 233 surgeons who were included in the study.

## Discussion

The NIH has created a new metric that provides field and time-normalized academic productivity assessments: the RCR. Bibliometric measurements benefit academia by identifying influential journals, articles, and authors.^10,11^ Within academic orthopaedics, the h-index and m-index have repeatedly been correlated with career duration and faculty rank, revealing their limitations in defining contemporary productivity.^12,13^ M-index is calculated by dividing a researcher's h-index by the span of active publication years. RCR provides a novel benchmarking method of comparing authors within their respective fields.^14^

To the author's knowledge, no study has conducted a comparative analysis between RCR and h-index among academic orthopaedic surgeons. It is well-known that factors such as sex and faculty rank are associated with historic measures such as h-index. By contrast, our study found a reduced effect of sex and career duration on m-RCR while w-RCR was consistent with the h-index. Furthermore, both w-RCR and h-index found higher faculty rank, higher number of fellowships, and longer career duration to be associated with higher scores. When exploring variables that contributed to a top quartile m-RCR score, sex, career duration, and number of fellowships were not notable contributors.

Regarding subspecialty differences, only minor divergence was found between these two metrics. H-index found the other subspecialty category to be the most productive while w-RCR found spine to be the most productive, followed by other; both metrics found pediatrics to be the least productive subspecialty. m-RCR found adult reconstruction to be the most productive subspecialty and musculoskeletal oncology to be the least. Similarly, a study conducted in 2021 found spine, upper extremity reconstruction, and adult reconstruction to have the highest h-indices.^15^ No literature exists about subspecialty differences in RCR within orthopaedics. Subspecialty differences were investigated to assess for potential confounding, not to conclude whether m-RCR or h-index was superior in this regard.

Recent changes in residency/fellowship support and mentorship programs for women have increased the attainment of surgical residencies.^16^ However, there remains a notable gender disparity among orthopaedic surgeons in our study. Of the women in this study, only 13.7% had more than 20 years in practice and 13.9% had full professorship status. Shorter career durations among women can most likely be attributed to a paucity of female orthopaedic surgeons before the past two decades. Despite having lack of leadership roles because of shorter career durations, a recent bibliometric analysis stated that there was no sex difference in m-index among academic orthopaedic surgeons, conflicting with prior literature on the h-index.^13,17^ Gender-specific data noted no difference in m-RCR (*P* = 0.066); however, it is important to mention that this *P*-value was relatively close to the accepted level of significance, so it cannot be said with certainty that m-RCR is superior to h-index for sex. Gender differences were noted in w-RCR (*P* < 0.001). Similar findings regarding sex and RCR were identified in an analysis of RCR among neurosurgeons, radiation oncologists, and plastic surgeons.^18-20^ m-RCR, a surrogate for research effect, may temporally adjust for differences in the sex composition of our sample. Perhaps, in the upcoming decades, this gender gap will narrow and w-RCR can become a more reliable metric in the assessment of productivity.

Career advancement and tenure are generally decided through three different tracks: clinical practice, research, and education.^21,22^ Research activities are commonly considered as the most influential for promotion.^23^ Career duration has served as a surrogate for faculty rank, as even in our study, each subsequent “years in practice” cohort had a greater percentage of professors. Although previous metrics have clearly delineated an association between career duration and faculty rank, this may not be so clear.^15,24^ Our findings suggest that those with 0 to 30 years in practice have no difference in effect. Effect only becomes notable after 30 years. A possible explanation for this may be that as academicians progress in their careers, they attain increased connections, resources, and skills, allowing them to have high-effect publications. Nonetheless, there does remain an effect of career duration on m-RCR. It is possible that m-RCR places younger authors at less of a disadvantage based on the gross number of articles published, potentially addressing a major criticism associated with the h-index.^5,9,25^ Using RCR, when assessing promotion, confers a benefit over historic solo metrics, such as the h and m-indices. Because m-RCR correlates with faculty rank, like the h-index, but not career duration, unlike the h-index, it should be considered as an optional novel metric for promotional assessment.

The h-index accounts for both the quantity and the scientific effect of the researcher's work, but the metric possesses several limitations.^5,9,25^ For instance, a few highly cited articles over one's entire career can markedly increase an author's h-index, which can be misleading. This can lead to a situation in which authors who are no longer proliferative in research retain a high h-index; the h-index does not account for changes in productivity or represent current productivity.^26^ Another clear limitation of h-index is unadjusted interfield variability because it does not allow for interfield comparisons. Finally, h-index has received criticism over its inherent bias toward older researchers; generally, these individuals have been active in research for longer periods, allowing for more accumulation of publications and citations. These described limitations can lead to an author's h-index being an inaccurate or misleading representation of productivity and effect.^1,5,9,25^

There are several limitations of RCR that should be addressed. These consist of lack of generalizability to international researchers, unacknowledged influence of author order and self-citations, and simplification of effect to number of citations. Nonetheless, RCR has multiple advantages over h-index, such as time and field normalization. m-RCR generates a more accurate depiction of effect by preventing gross number of publications from placing younger orthopaedic surgeons at a disadvantage.^5^

Because data collection for this study relied on publicly available residency program websites, missing data on faculty demographics is a limitation. However, the authors attempted to circumvent this by using medical networking services, such as Doximity and LinkedIn, to extract missing data elements. Nicknames or variation in last names can complicate author lookup. In addition, institutional websites can be outdated, so available information might not be completely accurate. There is also the possibility that certain programs or regions contain disproportionate amounts of missing data. Some orthopaedic surgeons belonged to multiple subspecialty cohorts because of completion of multiple fellowships. The NIH primarily provides funding to the United States, so the application of RCR may not apply internationally. Finally, self-citations were not investigated because neither the h-index nor RCR accounts for self-citations.

## Conclusion

Comprehensive bibliometric measurements, such as the h-index, m-index, and RCR, provide an objective representation of an academic surgeon's contribution to orthopaedic surgical research. While h-index is considered the current benchmark for measurement of academic productivity, our study demonstrates that using variations of RCR may sometimes be more effective and appropriate. H-index and w-RCR followed similar trends, reporting that men, along with higher faculty rank, higher number of fellowships, and longer career duration, are associated with higher productivity. However, m-RCR exhibits no differences based on sex or number of fellowships. m-RCR has the potential to reduce the historic bias against women and younger surgeons in orthopaedics, which has implications in employment, promotion, and tenure. Therefore, we propose that m-RCR can be used in conjunction with w-RCR or h-index to promote a fairer, comprehensive depiction of an orthopaedic surgeon's academic productivity.
